# Emerging Role of Transient Receptor Potential Vanilloid 4 (TRPV4) Ion Channel in Acute and Chronic Itch

**DOI:** 10.3390/ijms22147591

**Published:** 2021-07-15

**Authors:** Qiaojuan Zhang, Gwendolyn Henry, Yong Chen

**Affiliations:** 1 Department of Neurology, Duke University, Durham, NC 27710, USA; qiaojuan.zhang@duke.edu (Q.Z.); gwendolyn.henry@duke.edu (G.H.); 2 Department of Anesthesiology, Duke University, Durham, NC 27710, USA; 3 Department of Pathology, Duke University, Durham, NC 27710, USA

**Keywords:** TRPV4, itch, keratinocytes, sensory neurons, pruritogen

## Abstract

Itch is a clinical problem that leaves many sufferers insufficiently treated, with over 20 million cases in the United States. This is due to incomplete understanding of its molecular, cellular, and cell-to-cell signaling mechanisms. Transient receptor potential (TRP) ion channels are involved in several sensory modalities including pain, vision, taste, olfaction, hearing, touch, and thermosensation, as well as itch. Relative to the extensive studies on TRPV1 and TRPA1 ion channels in itch modulation, TRPV4 has received relatively little research attention and its mechanisms have remained poorly understood until recently. TRPV4 is expressed in ganglion sensory neurons and a variety of skin cells. Growing evidence in the past few years strongly suggests that TRPV4 in these cells contributes to acute and chronic disease-associated itch. This review focuses on the current experimental evidence involving TRPV4 in itch under pathophysiological conditions and discusses its possible cellular and molecular mechanisms.

## 1. Introduction

Itch (pruritus) is a sensory phenomenon characterized by an unpleasant sensation of the skin, triggering the desire to scratch [1]. Itch can be simply classified into acute and chronic itch, with the latter lasting longer than 6 weeks [2]. Acute itch serves an important protective function as a sentinel against potentially harmful external agents such as insects, toxic plants, and other irritants, while simultaneously removing the offending agent via scratching. On the other hand, chronic itch accompanies a number of skin diseases such as atopic dermatitis and dermatitis herpetiformis, as well as systemic conditions including hepatic cholestasis, diabetic neuropathy, kidney failure, and lymphomas [2,3]. Itch, particularly chronic itch, negatively impacts patients’ physical, social, and psychological well-being, leading to deterioration in their quality of life. Limited understanding of itch mechanisms hinders the development of effective antipruritic treatments.

Although itch and pain are distinct sensations, they share largely overlapping mediators, receptors, and neural circuits [4,5,6,7,8]. For example, many itch-inducing mediators, such as histamine, serotonin (5-HT), and endothelin-1 (ET-1), also cause pain [4]. Both itch and pain sensations are relayed by sensory neurons in the dorsal root ganglion (DRG) and trigeminal ganglion (TG). Whether itch and pain signals are actually transduced by the same populations of DRG neurons remains largely unexplored. Although there is increasing evidence that distinct subpopulations of sensory neurons are involved in transduction of pain and itch stimuli, a small subset of C-fiber nociceptors overlaps with pruriceptors [6]. Additionally, pain and itch employ largely overlapping transduction machinery, including transient receptor potential (TRP) ion channels, toll-like receptors (TLRs), and proteinase-activated receptors (PARs) [4]. TRP ion channels are involved in multiple sensory modalities, including pain [9]. Accumulating evidence has revealed that TRP ion channels are also implicated in the molecular mechanisms of itch [10,11,12,13], with experimental studies supporting significant roles for the chemo-irritant receptor TRPA1 mainly for non-histaminergic itch and the heat-capsaicin receptor TRPV1 largely for histaminergic itch. Unlike TRPA1 and TRPV1 channels, which are mainly expressed by primary sensory neurons, TRPV3, a warmth-activated TRP channel, is primarily expressed by skin keratinocytes and oral and nasal epithelia. Several lines of evidence suggest that TRPV3 is involved in itch [9,10,13,14]. TRPM8 is expressed in skin keratinocytes and a subset of primary afferent sensory neurons and serves as a cold sensor in vivo [9,10,14]. Under certain circumstances, cooling can relieve itch. Emerging evidence suggests that TRPM8 activation reduces itch [9,10,14]. Relative to ample evidence on other TRP ion channels, particularly TRPV1 and TRPA1, in itch [10,11,12,13], studies about TRPV4 remained scarce until recently.

TRPV4, a multimodally activated, nonselective cation channel, has been detected in sensory neurons of DRGs and TGs (DRG, TG) and skin cells (e.g., keratinocytes, mast cells, and macrophages) [9,15,16,17,18,19,20,21]. We recently reported TRPV4 as a critical component of the keratinocyte machinery that responds to ultraviolet B (UVB) radiation and functions critically to convert the keratinocyte into a pain-generator cell after UVB exposure [22]. One key mechanism in keratinocytes is increased expression and secretion of endothelin-1 (ET-1), which is also a known pruritogen [23,24,25], suggesting that TRPV4 may be involved in itch. Indeed, recent evidence from experimental and clinical-relevant studies has implicated that TRPV4 plays an important role in both acute and chronic itch. In this review, we will summarize current progress and elaborate on relevant highlights related to the cellular sites of action of TRPV4 in itch.

## 2. TRPV4 in Pruritogen-Evoked Acute Itch

Acute itch in the local affected skin is often caused by pruritogens (e.g., histamine, allergens, inflammatory mediators, and drugs). It is commonly experienced in daily life and has a protective role as to remove irritants and avoid further insults. Several studies demonstrated that TRPV4 contributes to pruritogen-evoked acute itch, although the results across laboratories are inconsistent (see Table 1). Akiyama et al. reported that *Trpv4* knockout (KO) mice displayed reduced scratching behavior in response to 5-HT, but not to histamine and SLIGRL (PAR-2 agonist). Interestingly, they found that chloroquine (CQ)-induced itch was even increased in *Trpv4* KOs [26]. In contrast, Kim et al. showed that both histamine- and CQ-induced itch was attenuated in mice lacking *Trpv4* [27]. Our group found that histamine-induced itch was decreased but CQ-induced itch remained unchanged in *Trpv4* KOs [17]. Further studies are warranted to clarify these discrepancies.

Although these inconsistent findings imply that TRPV4 plays a role in both histaminergic and nonhistaminergic itch, the crucial cellular site of action of TRPV4 remains obscure. The finding of TRPV4-dependent secretion of the pruritogen ET-1 by keratinocytes [22] led our group to question whether TRPV4 in keratinocytes of the epidermis can drive scratching behavior. To address this question, we used keratinocyte-*Trpv4* conditional knockout (cKO) mice to examine the specific contribution of keratinocyte TRPV4 to acute itch. In this study, an exciting new function of TRPV4 in forefront itch transduction from the integument was reported, namely that TRPV4 in epidermal keratinocytes functions as a pruriceptor-TRP channel in acute histaminergic (histamine and compound 48/80) itch, including itch evoked by ET-1, but not in non-histaminergic itch evoked by CQ [17]. Direct activation of TRPV4 with the selective agonist GSK101 also evoked scratching behavior that was dependent on TRPV4 expression in keratinocytes, thus underscoring the role of this cell and its expression of TRPV4 in itch. We further showed that Ca^2+^-transients in response to histamine, 48/80, and ET-1 in cultured primary keratinocytes depend on TRPV4, and Ca^2+^ influx via TRPV4 increased phosphorylation of extracellular signal-regulated kinase (p-ERK) in keratinocytes. Consequently, topical transdermal treatment with TRPV4 selective inhibitor GSK205 was effective as an anti-pruritogen. Moreover, we found similar in vivo anti-pruritic effects when topically targeting mitogen-activated protein kinase (MEK), upstream of ERK, with the selective inhibitor U0126 [17]. We also employed sensory neuron-*Trpv4* cKO mice and determined the specific contribution of sensory neuron TRPV4 to acute itch behaviors in mice. Our results show that TRPV4 in sensory neurons contributes to scratching behavior evoked by histaminergic (histamine and 48/80) and partial histaminergic (5-HT), but not non-histaminergic (SLIGRL and CQ) pruritogens [28]. Together, these studies suggest that both skin keratinocytes and sensory neurons are important locales where TRPV4 differentially regulates various forms of acute itch.

Conditional KO of *Trpv4* in skin keratinocytes [17] or sensory neurons [28] significantly attenuated histamine-induced scratching behavior, which is in line with Kim et al. [27] and our studies [17] but in contrast to Akiyama’s finding using *Trpv4* KOs [26]. Although CQ-induced itch has been reported as increased, decreased, or unchanged in *Trpv4* KOs, we found that it was not significantly altered in either sensory neuron- or skin keratinocyte-*Trpv4* cKOs. In support of Akiyama’s report, we observed that cKO of neuronal *Trpv4* significantly reduced 5-HT, but not SLIGRL-evoked itch [28]. The differences in certain types of acute itch between *Trpv4* KO and sensory neuron- or keratinocyte-*Trpv4* cKO mice might be due to the potential gene-regulatory developmental compensation and the possible contributions to itch of TRPV4 in other cell lineages, such as skin macrophages, mast cells, and endothelial cells.

Clinical evidence suggests that thermal changes of skin can modulate itch sensation. At the molecular level, temperature sensation involves thermosensitive TRP ion channels. One of these, TRPV4, can be activated by innocuous warmth [29]. Interestingly, the contribution of warm temperatures to itch perception via keratinocyte TRPV4 has been demonstrated: Sanders et al. recently reported that innocuous warmth of the skin significantly increased 5-HT-evoked scratching and spontaneous scratching in the ovalbumin model of atopic dermatitis but decreased histamine-evoked scratching, which was blocked by TRPV4 selective antagonist GSK205 [30].

Cinnamaldehyde (CA), the TRPA1 selective agonist, was reported to elicit an itch sensation when applied topically to human skin [31]. It was reported that CA elicits significantly fewer scratch bouts in *Trpv4* KO mice, compared with WTs [32]. In Ca^2+^ imaging studies, CA excited 24% of DRG neurons of WT mice, but significantly fewer in neurons from *Trpv4* KOs. In contrast to the reduced scratching upon i.d. injection of CA, alloknesis scores (itch due to mechanical stimuli) following topical application of CA were significantly higher in *Trpv4* KOs. It remains unknown why alloknesis is increased in *Trpv4* KOs, but it may reflect a developmental compensatory increase in TRPA1 expression in neurons of mice lacking *Trpv4*.

## 3. TRPV4 in Experimental Chronic Itch

Chronic itch has long been known not only as a concomitant manifestation of various skin diseases, such as contact dermatitis, eczema, atopic dermatitis, psoriasis, etc., but also occurs in systemic, neurological, or psychiatric diseases [2,3]. Acute itch has a protective role by removing irritants to avoid further damage, yet chronic itch is debilitating and negatively impacts one’s quality of life. Recent evidence strongly suggests that TRPV4 contributes to chronic itch (see Table 2).

**Figure 1:**
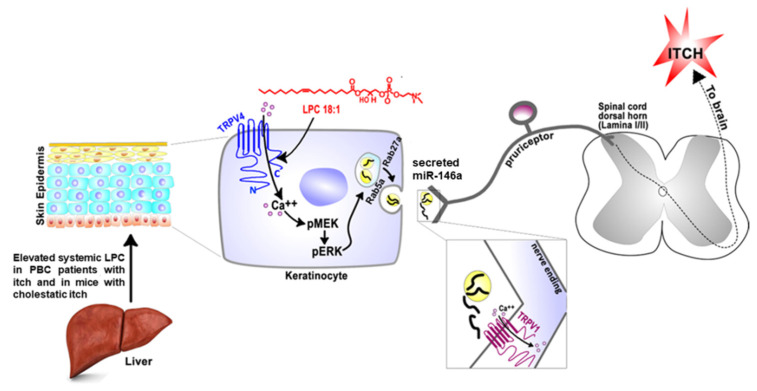
Schematic diagram depicting the potential mechanism underlying cholestatic itch. Cholestatic liver disease is associated with significantly elevated systemic LPC, which directly activates TRPV4 expressed in skin keratinocytes. This in turn leads to extracellular release of miR-146a via MEK–ERK–Rab5a/Rab27a signaling pathways. miR-146a functions as a pruritogen by activating TRPV1-expressing pruriceptor sensory neurons that innervate the skin. Activation of TRPV1 by miR-146a induces the sensation of itch via central pathways.

Cholestatic itch has significant prevalence in patients with hepatobiliary diseases: namely primary biliary cholangitis (PBC), primary sclerosing cholangitis (PSC), and intrahepatic cholestasis of pregnancy (ICP) [33]. Therapeutic recourse is dire because the underlying pathophysiology remains largely elusive. Cholestatic itch has been linked to bile acids, bilirubin, progesterone metabolites, and lysophosphatidic acid (LPA) [34,35,36,37,38,39]. LPA is a bioactive phospholipid with diverse biological functions [40]. Autotaxin (ATX) catalyzes the hydrolysis of lysophosphatidylcholine (LPC) to LPA, and it has been demonstrated that levels of ATX and LPA are elevated in patients with cholestatic liver disease [34]. LPC, the precursor of LPA, has been linked with a variety of diseases that are pruritic. For instance, previous studies demonstrated that LPC concentrations are increased in blood [41] and lesional skin [42] of psoriasis patients. Mass spectrometry of lesional skin from atopic dermatitis patients revealed an increase in short-chain LPC species [43]. Moreover, a recent study found that LPC concentrations are significantly elevated systemically in uremic patients with pruritus [44]. In our recent study [16], we demonstrated that LPC, as a novel pruritogen, is robustly pruritic in mice, and that TRPV4 in skin keratinocytes is essential for LPC-induced itch and itch in mice with cholestasis in a model of α-naphthyl-isothiocyanate (ANIT). Three-dimensional structural modeling, site-directed mutagenesis, and channel function analysis suggested a TRPV4 C-terminal motif for LPC binding and channel activation. In keratinocytes, TRPV4 activation by LPC induced extracellular release of microRNA-146a (miR-146a), which activated TRPV1^+^ sensory neurons to cause itch. Both LPC and miR-146a levels were elevated in sera of primary biliary cholangitis patients with itch and correlated with itch intensity. Moreover, LPC and miR-146a were also increased in sera of cholestatic mice and elicited itch in nonhuman primates. Our findings suggest a hitherto underappreciated function of the epidermis and of TRPV4 in keratinocytes as a key signaling molecule in cholestatic itch, as summarized in Figure 1.

Dry skin is caused by reduction in water-holding capacity, which is regulated by cutaneous barrier function in the stratum corneum. Some reports implicated skin dryness itself and/or cutaneous barrier disruption in dry skin-associated pruritus [45,46]. It has been suggested that TRPV4 contributes to skin barrier recovery [47] and intercellular junction formation in skin keratinocytes [20], as well as increases in Ca^2+^ that are critical for accelerating barrier recovery after stratum corneum disruption [21,48]. Using a dry-skin model of topical application of acetone/ether mixture followed by water (AEW), Luo et al. found that AEW treatment increases TRPV4 expression in the skin and scratch bouts are markedly reduced in *Trpv4* KO mice. Furthermore, AEW-induced itch was attenuated by the TRPV4 selective inhibitor HC067047 when applied either systemically or topically [19]. The latter indicates that TRPV4 in skin cells may be required for itch associated with dry skin. To determine the cellular site of action, they found that the AEW-induced spontaneous scratching response is significantly reduced in keratinocyte *Trpv4* cKOs, but not in mice lacking macrophage *Trpv4*. With the same model, we found that *Trpv4* deficiency in sensory neurons also leads to a reduction in AEW-induced itch [28]. Together, these results imply TRPV4 in both skin keratinocytes and sensory neurons contributes to dry- skin-associated itch.

Allergic contact dermatitis (ACD) is a skin T-cell-mediated immune response caused by non-toxic allergens in contact with the skin, and it is often accompanied by debilitating itch [49]. Several groups reported that TRPV4 in skin is upregulated in ACD models induced by squaric acid dibutylester (SADBE) [19,50] or 2,4-dinitrofluorobenzene (DNFB) [51], implicating TRPV4 might contribute to ACD-associated itch. Similar to what they found with the dry skin model, Luo et al. reported that *Trpv4* KOs display a markedly reduced scratching response after SADBE treatment when compared with WT mice. Systemic or topical administration of HC067047 significantly reduced spontaneous scratching bouts in WT mice treated with SADBE. Furthermore, they found that the SADBE-induced spontaneous scratching response is significantly reduced in macrophage *Trpv4* cKOs, but not in mice lacking keratinocyte *Trpv4* [19]. These studies suggest that TRPV4 function in macrophages and keratinocytes contributes differentially to the pathogenesis of allergic (ACD) and nonallergic (dry skin) chronic itch in mice. In contrast to the functional role of TRPV4 in keratinocytes, we did not find a significant contribution of TRPV4 in sensory neurons to scratching behaviors in an ACD model of DNFB [28]. Together, these findings indicate that ACD-associated itch might rely on TRPV4 in peripheral skin cells, but not sensory neurons.

Psoriasis is a chronic inflammatory skin disease affecting up to 2–3% of the world’s population, and the majority of psoriasis patients suffer from itch [52]. Yan et al. reported that TRPV4 in DRGs and skin is upregulated at both gene and protein levels in a mouse model of psoriasis, induced by topical application of imiquimod (IMQ) [53]. With the same animal model, Akiyama et al. reported that TRPV4 at the mRNA level is increased in DRGs, but decreased in skin [54]. Although there is some inconsistency regarding TRPV4 expression in skin after psoriasis between these two studies, an in vivo behavioral study demonstrated that IMQ-induced psoriatic itch is reduced in *Trpv4* KO mice [53].

## 4. TRPV4 in Itch: Evidence from Human Studies

Several clinical-relevant studies have supported TRPV4 playing a role in the pathogenesis of chronic itch by demonstrating differential expressions of TRPV4 in chronically pruritic skin. Luo et al. reported a significant increase in *Trpv4* mRNA in skin biopsies of chronic idiopathic pruritus patients in comparison with healthy controls [19]. Pruritus following burns is common, with mild to severe pruritus in 87% of patients at 3 months, 70% at 12 months, and 67% at 24 months after burn injury [55]. Yang et al. reported that there is a trend of increased TRPV4-immunoreactive expression in burn scars of patients with pruritus versus without pruritus. In addition, a significant increase in *Trpv4* mRNA was detected in burn scars of patients with pruritus and the level of *Trpv4* mRNA was positively correlated with the intensity of pruritus [56]. Using RNA sequencing, Nattkemper et al. found there was no significant difference in the levels of *Trpv4* mRNA in pruritic, lesional skin versus non-pruritic, non-lesional skin for neither atopic dermatitis nor psoriasis patients, but there was a significant increase in pooled pruritic, lesional skin of atopic dermatitis and psoriasis patients versus healthy controls [57]. Interestingly, a recent study demonstrated that the expression levels of TRPV4 in the skin are positively correlated with the severity of the psoriasis, yet the itch phenotypes were not assessed in these patients [53]. Chronic itch may be accompanied by other sensory symptoms. For example, some patients with chronic itch report a warm sensation, while others describe stinging or burning [58,59,60]. Hidding et al. found that chronic itch patients experiencing neuropathic symptoms had significantly higher epidermal TRPV4 expression compared to the healthy controls [61]. These patients displayed an increased response to capsaicin (TRPV1 agonist), including stinging or burning sensations. It is possible that increased TRPV4 expression in skin cells might trigger the release of some substances (possibly proteins, peptides, and phospholipids), which can sensitize TRPV1 ion channel in sensory neurons via direct keratinocyte–nerve fiber signaling or indirect involvement of immune, vascular, and other adjacent cells, leading to an increased response to capsaicin in such patients. Further studies are warranted to investigate this possibility.

## 5. Conclusions

In summary, we have reviewed several lines of evidence on TRPV4 in acute and chronic itch, mainly focusing on the functional contribution of TRPV4 in skin cells and sensory neurons to itch. Chronic itch is one of the most debilitating human diseases and represents a social and economic burden for our society. Considering that the current therapies often have unsatisfactory efficacy, finding more effective treatments for chronic itch is a high priority. Summarizing the findings of TRPV4 in itch may provide a better understanding of this debilitating disease and a rationale for the development of new anti-itch treatments. Although studies conducted over the past few years strongly indicate that TRPV4 contributes to itch, some important questions remain open to be discussed and resolved. For example, the findings across different laboratories are inconsistent for some forms of itch evoked by pruritogens (see Table 1) and more studies are needed to fully understand TRPV4′s role in acute itch. Although it was reported that TRPV4 is expressed in skin mast cells [15] and spinal cord astrocytes [62], both known to be involved in itch [8,63], whether TRPV4 in these cells modulates itch remains elusive. It has been suggested that TRPV4 and TRPV1 can possibly form a heteromeric complex in DRG neurons and the function of TRPV4 in acute itch involves TRPV1-mediated facilitation [27]. Further studies are needed to elucidate whether the interaction of TRPV4 with TRPV1 in sensory neurons plays a role in chronic itch and whether this interaction in skin cells (e.g., keratinocytes) contributes to itch. Although these additional studies are warranted, it can possibly be proposed that combined approaches simultaneously targeting these channels may prove advantageous in the management of pruritus. While some questions remain to be answered in future studies, it should be reiterated that current evidence strongly suggests that TRPV4 in skin cells (e.g., keratinocytes and macrophages) and DRG sensory neurons contributes to itch. This concept bears the possible translational medical relevance to develop selective anti-TRPV4 treatments for itch disorders. For example, a potential trial could be topical application of the TRPV4 selective inhibitor for treating chronic itch, based on recent exciting findings about keratinocyte TRPV4 in cholestatic itch [16] and dry-skin-associated itch [19]. Although off-target effect is a potential roadblock for TRPV4 as a candidate for an anti-itch drug given its wide expression and various biological functions, TRPV4 may hold promise for the treatment of itch disorders.

## Figures and Tables

**Table 1:** Effects of global or conditional knockout of *Trpv4* on pruritogen-induced acute itch behaviors.

Pruritogen-Evoked Acute Itch	*Trpv4* KO	Keratinocyte-*Trpv4* cKO	Sensory Neuron-*Trpv4* cKO	Macrophage-*Trpv4* cKO
Histamine	Unchanged [26] or attenuated [17,27]	Attenuated [17]	Attenuated [28]	Unknown
Compound 48/80	Attenuated [17]	Attenuated [17]	Attenuated [28]	Unknown
ET-1	Attenuated [17]	Attenuated [17]	Unknown	Unknown
5-HT	Attenuated [26]	Unknown	Attenuated [28]	Unknown
SLIGRL	Unchanged [26]	Unknown	Unchanged [28]	Unknown
Chloroquine	Increased [26], unchanged [17] or attenuated [27]	Unchanged [17]	Unchanged [28]	Unknown

**Table 2:** Effects of global or conditional knockout of *Trpv4* on experimental chronic itch behaviors.

Chronic Itch Model	*Trpv4* KO	Keratinocyte-*Trpv4* cKO	Sensory Neuron-*Trpv4* cKO	Macrophage-*Trpv4* cKO
Dry skin model (AEW)	Attenuated [19]	Attenuated [19]	Attenuated [28]	Unchanged [19]
Allergic contact dermatitis models (SADBE or DNFB)	Attenuated [19]	Unchanged [19]	Unchanged [28]	Attenuated [19]
Cholestatic itch model (ANIT)	Unknown	Attenuated [16]	Unknown	Unknown

## Data Availability

Not applicable.
